# Fish and chips: Using machine learning to estimate the effects of basal cortisol on fish foraging behavior

**DOI:** 10.3389/fnbeh.2023.1028190

**Published:** 2023-02-08

**Authors:** Wallace M. Bessa, Lucas S. Cadengue, Ana C. Luchiari

**Affiliations:** ^1^Department of Mechanical and Materials Engineering, University of Turku, Turku, Finland; ^2^Programa de Pós-Graduação em Engenharia Elétrica e de Computação, Universidade Federal do Rio Grande do Norte, Natal, Brazil; ^3^Departamento de Fisiologia, Centro de Biociências, Universidade Federal do Rio Grande do Norte, Natal, Brazil

**Keywords:** foraging, fish, multi-armed bandit, epsilon-greedy, cortisol

## Abstract

Foraging is an essential behavior for animal survival and requires both learning and decision-making skills. However, despite its relevance and ubiquity, there is still no effective mathematical framework to adequately estimate foraging performance that also takes interindividual variability into account. In this work, foraging performance is evaluated in the context of multi-armed bandit (MAB) problems by means of a biological model and a machine learning algorithm. Siamese fighting fish (*Betta splendens*) were used as a biological model and their ability to forage was assessed in a four-arm cross-maze over 21 trials. It was observed that fish performance varies according to their basal cortisol levels, i.e., a reduced average reward is associated with low and high levels of basal cortisol, while the optimal level maximizes foraging performance. In addition, we suggest the adoption of the epsilon-greedy algorithm to deal with the exploration-exploitation tradeoff and simulate foraging decisions. The algorithm provided results closely related to the biological model and allowed the normalized basal cortisol levels to be correlated with a corresponding tuning parameter. The obtained results indicate that machine learning, by helping to shed light on the intrinsic relationships between physiological parameters and animal behavior, can be a powerful tool for studying animal cognition and behavioral sciences.

## 1. Introduction

Foraging plays an essential role in animal's fitness and its efficacy has been shaped by evolutionary processes (Pearson et al., 2014). However, in addition to its phylogenetic basis, foraging behavior is also largely influenced by ontogeny (Hughes et al., 1992; Grecian et al., 2018). In fact, both intrinsic and extrinsic factors that modulate behavior confer different consequences on fitness (Dingemanse et al., 2004; Brown et al., 2007) and the huge variation between individuals' life history leads to several differences in the way they deal with stressful situations, how sensitive to changes they are, or even how they learn new tasks.

But how do physiological responses correlate with these factors and their corresponding behavioral patterns? In this context, Koolhaas et al. (1999) defined two main profiles: proactive individuals show high exploration, are less sensitive to environmental changes, have low latency to aggression and low hypothalamic-pituitary-adrenal (HPA) response, while reactive are less explorative, highly dependent on environmental stability, have less routine development and high HPA reactivity. Based on an extensive literature review, Toscano et al. (2016) suggested that these behavioral traits and their interindividual variations can lead to specialization in foraging behavior. Cortisol levels are indeed highly influential on many traits, including immune response (Zhang et al., 2022), reproductive investment (Jiang et al., 2022), metabolism (Fernandes Silva et al., 2022; Winberg and Sneddon, 2022), energy reallocation to cope with stressors (Gorissen and Flik, 2016), and the behavioral and cognitive responses (Alfonso et al., 2019, 2020; Torgerson-White and Sánchez-Suárez, 2022). Furthermore, it is also important to emphasize that in many animal species, basal and post-stress cortisol levels, as well as behavioral responses, are correlated in such a way that these characteristics are used to identify coping styles (Alfonso et al., 2020).

Recently, Morimoto (2019) showed that foraging decisions can be represented in the context of multi-armed bandit (MAB) problems. The MAB problem is a machine learning framework used to investigate the exploration vs. exploitation dilemma: the learning agent should try to maximize the cumulative reward by choosing between exploitation (an immediate payoff based on available information) or exploration (looking for options that may lead to a better payoff). Reinforcement learning algorithms proposed to deal with this problem usually rely on a parameter to modulate the agent exploitation/exploitation tradeoff (Sutton and Barto, 2018). Moreover, it is already known that different species have developed different strategies to balance exploration and exploitation depending on the ecological conditions of their environment. Within the same species, these strategies may be modulated by specific internal states and their corresponding physiological parameters. Therefore, assuming that interindividual physiological differences lead to distinct foraging behaviors, would it be possible to estimate foraging performance based on physiological indicators?

In this work, we propose that basal cortisol can be used as a predictor of the foraging behavior of the Siamese Fighting Fish, *Betta splendens*, as well as its performance in MAB related tasks. Despite their ancient evolutionary appearance, fishes stand for good biological models. Their brain structure and rich behavioral repertoire are comparable to those of mammals (Gerlai, 2014), which offers many possibilities for research on vertebrate evolution, including both cognitive and behavioral comparative analyses. To model foraging decisions, we apply the ε-greedy algorithm (Sutton and Barto, 2018) and suggest that the parameter ε, used to modulate the exploration/exploitation tradeoff, correlates with normalized basal cortisol levels of fish. The close agreement between the results obtained with biological and computational models also allows us to suggest that simple algorithms like this one can not only describe quite complex behavioral patterns, but also characterize the decision mechanisms of many organisms.

## 2. Materials and methods

### 2.1. Biological model

Eighty-six male *Betta splendens* (adults, 3 months old, acquired in a local breeding facility in Natal/RN) were kept in individual tanks (3L, 15 × 15 × 15cm^3^) in the vivarium of the Fish Laboratory at the Federal University of Rio Grande do Norte throughout the experimental phase. They were weighed and measured (0.87 ± 0.15g and 2.70 ± 0.21cm) before being placed in the tanks. The tanks were arranged side by side with an opaque partition between them to avoid visual contact between the fish. The water in the tanks was changed every 2 days and its quality was monitored daily: 28 ± 1°C, pH~6.7 and O_2_~6mg/L. The photoperiod was set on a 12:12 light/dark cycle, and the animals were fed *ad libitum* twice a day with commercial pellet food (Alcon Betta, 44% protein and 5% fat) and frozen *Artemia salina*.

In the foraging task, a cross-maze was used: 10cm high, with a central area of 10 × 10cm^2^, and four arms measuring 30cm long by 10cm wide each. Each fish was individually placed in the central area, in a lift-up start box, for 60 s and then released to explore the maze. One of the cross-maze arms had a small red circular plate (3cm diameter) on which five units of *Artemia salina* were placed when fish entered the area (reward area). The reward arm was randomly chosen for each trial to prevent the fish from learning any other cues other than the red circular plate. This procedure was performed 21 times (trials) over 11 days. A reward equal to one was assigned to each trial in which the fish entered first the arm with the circular plate (success), and equal to zero in the other cases (failure). There were no cues on the maze walls but the feeding plate. A black curtain was used to hide the researcher from the fish during the trials. After each trial, the fish was removed from the maze and returned to its original tank. All trials were recorded using a camcorder (JVC Full HD 60x, model GZ-HM65BU) located above the tank.

To estimate basal cortisol levels, the fish were euthanized 24 h after the last trial, all at the same time, with a high dose of anesthetic (clove oil, 10mL/L) and stored in individual Falcon tubes at −20°C. Each whole-body sample was macerated with 3mL of phosphate-buffered saline (PBS) using a high-speed stirrer (Nova Tecnica Homogenizador Potter NT 136). The resulting solution was then centrifuged (e.g., for 10 min at 3000g and 7°C). To standardize fish samples, 1dL aliquots of supernatant from each fish homogenate were collected and transferred to Eppendorf tubes. A salivary cortisol ELISA kit (SLV 2930 Lot 64K044 DRG Diagnostics) was used. Both cortisol extraction and kit validation followed Sink et al. (2007). The cortisol test failed for three individuals and for another one it presented an outlier, resulting in 82 valid measurements. Moreover, to facilitate comparative analysis, the cortisol levels were rescaled to range from zero to one using min-max normalization and then the 82 fish were divided into five groups (2 with 17 individuals, 3 with 16 individuals). Thus, in order to allow a better visualization of the obtained results and facilitate the identification of different foraging patterns, the fish were divided into two groups with low levels of basal cortisol, two with high levels and an intermediate group. Table 1 shows the fish groups with their corresponding sample sizes, measured cortisol ranges (in ng/mL) and normalized values (group means with standard errors). An OpenDocument Spreadsheet with the foraging data is available as Supplementary material.

**Table 1:** Fish groups divided by basal cortisol level.

**Group**	**Sample size**	**Cortisol range (in ng/mL)**	**Normalized cortisol (mean ±SE)**
1	16	1.68–10.69	0.0289 ± 5.22E-03
2	17	12.78–28.55	0.1413 ± 9.85E-03
3	16	32.18–87,35	0.4103 ± 4.32E-02
4	16	90.50–117.33	0.8137 ± 1.53E-02
5	17	117.44–135.40	0.9251 ± 9.74E-03

### 2.2. Computational model

The ε-greedy algorithm relies on a single parameter ε to define the exploratory bias. The parameter can range from 0 to 1, which directly corresponds to a continuum from high exploitation to high exploration. At each iteration (trial), the algorithm chooses between exploiting (with probability 1−ε) the best arm so far or exploring (with probability ε) a new one. After choosing an arm *A*, the algorithm assigns the corresponding reward *R* to this action, updates the number of times this arm was visited *N*(*A*) and its action-value *Q*(*A*). The value *Q*(*A*) of an arm *A* represents the mean reward for this action up to the current trial. After visiting the arm in a trial and getting the corresponding reward, the action-value is updated as follows Sutton and Barto (2018):


(1) $$Q(A)\leftarrow Q(A)+\frac{1}{N(A)}[R-Q(A)].$$

The ε-greedy algorithm for a bandit with *k* arms is presented in the box below and its implementation in Python is available as Supplementary material. In the simulation studies, to replicate the foraging task, four arms were considered *a* = [1, 2, 3, 4] but only one of them yielded a reward *R* = 1 (for the other three arms there was no payoff, *R* = 0). All fish had their computer versions properly run, with each epsilon being assigned the corresponding normalized cortisol level. Two sets of simulations were performed for the 82 epsilons: first with 21 trials for each ε (for comparative analysis with fish) and then with 400 trials (to assess their long-term performance).

**Algorithm 1:**
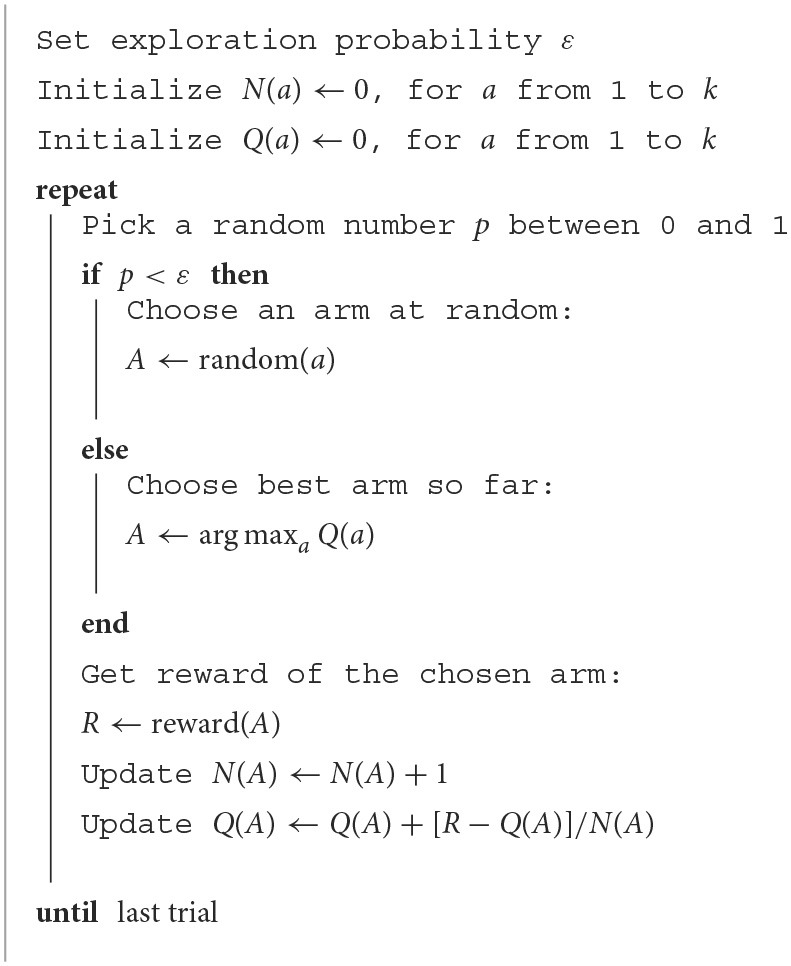
ε-greedy for a *k*-armed bandit

## 3. Results and discussion

**Figure 1:**
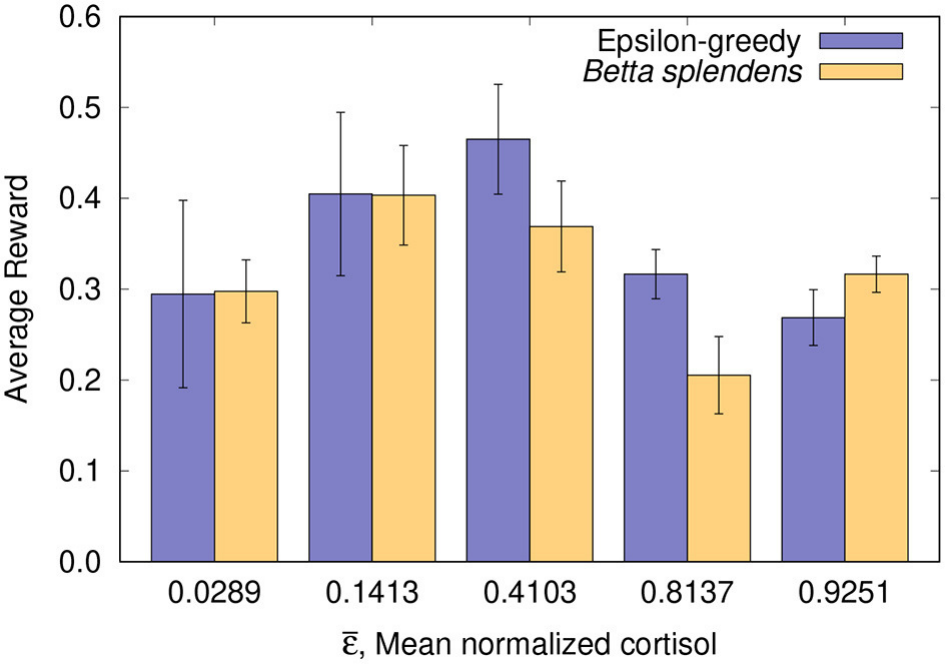
Foraging performance after 21 trials: average rewards (mean±SE) from both fish and simulation groups organized by mean normalized cortisol or equivalent mean epsilon $\bar{\varepsilon }$.

The foraging performance of both biological and computational models after 21 trials is shown in Figure 1 and Table 2. The data (mean±SE) are presented for each group based on the average rewards obtained by the individuals. For each individual, the average reward stands for the sum of its obtained payoffs divided by the maximum possible score (21 in this case).

**Table 2:** Average rewards from both fish and simulation groups after 21 trials.

**Group**	**ε-greedy (mean±SE)**	***Betta splendens* (mean±SE)**
1	0.2946 ± 0.1031	0.2976 ± 0.0346
2	0.4048 ± 0.0899	0.4034 ± 0.0548
3	0.4650 ± 0.0605	0.3690 ± 0.0500
4	0.3165 ± 0.0271	0.2054 ± 0.0425
5	0.2687 ± 0.0307	0.3165 ± 0.0200

It can be seen in Figure 1 and Table 2 that the results obtained with the fish and with the corresponding computer simulations are in close agreement, which allows us to suggest that the ε-greedy algorithm is indeed capable of estimating the foraging performance of the Siamese Fighting Fish, *Betta splendens*. Moreover, it also supports the assumption that the algorithm can be used to investigate the effects of physiological parameters on foraging behavior. By observing the results in Figure 1, it can be verified that the normalized cortisol level and the parameter ε show a good correlation with respect to the average reward obtained in the corresponding groups. In both cases, a reduced average reward is associated with low and high values of ε (or the normalized basal cortisol, in the biological model), while an optimal value maximizes foraging performance. In fact, this variability can be explained from the algorithm's point of view, since ε is linked to exploratory bias, and its optimal value is related to the best exploration/exploitation tradeoff for each foraging task.

As expected, extreme levels of basal cortisol, both on the right and on the left, lead to a lower foraging performance, according to an inverted-U pattern. This inverted-U shape is commonly seen in the relationship between glucocorticoids and performance in cognitive functions such as learning and decision making (Salehi et al., 2010; Schilling et al., 2013). As a matter of fact, both low and high cortisol levels are linked to cognitive dysfunction (Maripuu et al., 2014). It has been shown that cases of major depression correlate with a very low level of cortisol (Keilp et al., 2016), which cannot stimulate normal body and brain functioning, including motivation. On the other hand, high cortisol levels are related to chronic stress, and also associated with poor performance (Lupien et al., 2018). Thus, inspecting the cortisol data, such variation was already expected, as well as its relationship with the performance of the fish in the learning task. The biological relevance here concerns the ability of animals to learn and respond appropriately to environmental demands, i.e., animals that are in a situation of low or high levels of cortisol (derived from some previous situation experienced by them) may present performance impairment and threat to fitness.

Like cortisol, other factors that influence foraging can affect the animal's ability or the way it learns, for example, the speed to make associations (Wang et al., 2015). As suggested by Raoult et al. (2017), low-stress fish show higher levels of activity and explore the environment more than high-stress fish, which leads to learning the reward location as a result of their proactive coping style (Koolhaas et al., 1999).

It should be noted that the slight discrepancies observed for intermediate and higher values of mean epsilon/cortisol in Figure 1 and Table 2 are likely due to the low number of trials, which can lead to a greater performance variability, especially in cases where a more exploratory behavior is involved. Moreover, in Group 4 (with corresponding mean normalized cortisol of 0.8137) it happened that 25% of the individuals (4 out of 16) did not accumulate any reward, which ended up reducing the average reward of the entire group and increasing its discrepancy in relation to computer estimate. For comparison purposes, in the other groups, at most one fish did not accumulate a reward, which led to a closer agreement between the data obtained with the fish and the algorithm. In order to make this relationship even clearer, the correlation coefficient between the data obtained with the fish and with the epsilon-greedy algorithm can be calculated for two different scenarios:

1. With all groups included, where *r* = 0.62 (moderately correlated) is obtained;
2. With all groups except Group 4, where *r* = 0.81 (highly correlated) is obtained.

As a matter of fact, it is known that foraging performance can be improved through learning, which in fish is most easily measured by the number of exposures required (Warburton, 2003). However, this learning efficiency is heavily influenced by the complexity of the task, which can make the minimum number of trials range from < 10 to more than 200 to reach an asymptote (Hughes et al., 1992). For practical reasons, associative tasks involving fish models are usually repeated 20 times (Luchiari et al., 2015) of which significant behavioral changes (learning) begin to occur around the 12^th^ trial. In addition, internal factors such as hormone levels also affect the animals' performance.

**Figure 2:**
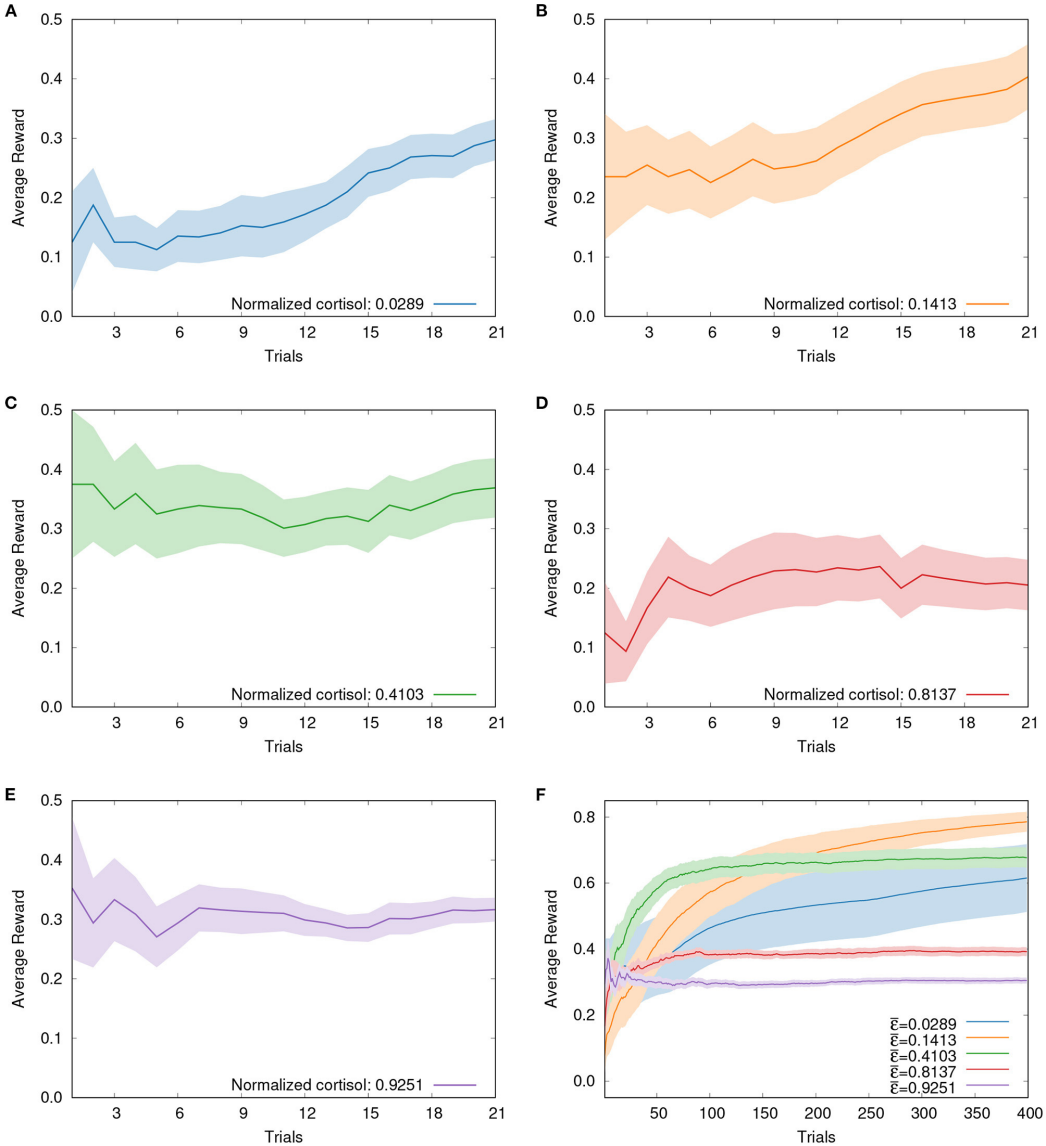
Evolution of cumulative rewards for both fish and simulation groups: solid lines depicting mean values and shaded areas representing the standard errors. **(A)** Fish from Group 1 (mean cortisol of 0.0289) over 21 trials. **(B)** Fish from Group 2 (mean cortisol of 0.1413) over 21 trials. **(C)** Fish from Group 3 (mean cortisol of 0.4103) over 21 trials. **(D)** Fish from Group 4 (mean cortisol of 0.8137) over 21 trials. **(E)** Fish from Group 5 (mean cortisol of 0.9251) over 21 trials. **(F)** Five simulation groups over 400 trials. Observing the evolution of payoffs, two different patterns of reward accumulation can be clearly identified: while for lower values of cortisol payoffs increase progressively throughout the trials, as can be seen in **(A, B)**, for intermediate and high values the cumulative rewards reaches a stable plateau more quickly, as shown in **(C–E)**. Long-term simulation results presented in **(F)** confirm these trends observed with fish.

Furthermore, if the evolution of cumulative rewards over trials is inspected, two distinct patterns can be clearly observed. The results (mean±SE) obtained with *Betta splendens* and ε-greedy are presented in Figure 2. In Figures 2A, B, for instance, it can be seen that for lower values of basal cortisol the payoffs increase progressively over the trials, while for intermediate and high values the cumulative rewards reaches a stable plateau more quickly, as shown in Figures 2C–E. The ε-greedy algorithm in this context can also help to confirm this trend in the long term (see Figure 2F). The simulation of the five groups along 400 trials shows that the best foraging performance is shifted to the left as the number of trials increases. That is, if we select specific numbers of trials, we will see that the best foraging performance, as the number of trials increases, will gradually be obtained by individuals with lower levels of basal cortisol. This suggests that the exploratory behavior associated with higher basal cortisol levels is initially favored, but as the number of trials increases, individuals with lower basal cortisol levels, i.e., prone to exploitation, tend to perform better. Further experiments involving more trials, different types of mazes and even other biological models are expected to corroborate this conjecture.

It is worth mentioning that our biological results echo work on individuals' differences in speed-accuracy tradeoffs (Raoult et al., 2012, 2017; Mamuneas et al., 2015), indicating that corticosteroids modulate the learning performance. It also suggests that individuals with different basal corticoid levels employ distinct learning strategies and may need different number of trials to reach the same level of performance.

Moreover, our findings endorse that multi-armed bandits can be a reasonable framework to evaluate foraging decisions, as suggested by Morimoto (2019). We show that the ε-greedy turns out to be a simple tool but capable of describing both qualitatively and quantitatively the animal behavior in foraging tasks. By means of a stochastic approach, the algorithm allows the exploratory behavior to be uniformly spread across all trials. This is an essential feature to capture the ability of animals to make decisions in an ever-changing dynamic environment.

Finally, it is important to highlight that this synergy between computer and biological sciences can benefit both areas. In fact, much of the work done on machine learning has been guided by the research on animal cognition and behavior. Biologically inspired schemes have indeed allowed the development of more efficient algorithms for autonomous robots (Bessa et al., 2017, 2018). Furthermore, considering that decision-making algorithms are already quite widespread in our society, with applications ranging from targeted advertisements and recruitment choices to medical diagnoses and prison sentences, it is crucial and urgent to better understand their capabilities and limitations. In this sense, animal behavior can provide powerful insights and end up helping to design more efficient and ethical algorithms.

## Data availability statement

The original contributions presented in the study are included in the article/Supplementary material, further inquiries can be directed to the corresponding author.

## Ethics statement

The animal study was reviewed and approved by Ethics Committee for Animal Use of the Federal University of Rio Grande do Norte (CEUA-006/2016).

## Author contributions

WB, LC, and AL contributed to conception and design of the study. AL organized the fish database. LC implemented the computer code and performed the computational analysis. WB and AL wrote the first draft of the manuscript. All authors contributed to manuscript revision, read, and approved the submitted version.

## Supplementary material

The OpenDocument Spreadsheet with the foraging data and the Python implementation of the ε-greedy algorithm for a bandit with *k* arms are available as Supplementary material.
