# Supplementary material for: Fish and chips: Using machine learning to estimate the effects of basal cortisol on fish foraging behavior
Source: Front Behav Neurosci. 2023 Feb 8;17:1028190. doi: 10.3389/fnbeh.2023.1028190 (PMC9944048; doi:10.3389/fnbeh.2023.1028190)
Supplement: Supplementary file 1 [file Data_Sheet_1.PDF]

```python
# -*- coding: utf-8 -*-
"""
Created on Fri Mar 29 15:37:48 2019

@author: lucas
"""
"""
This function implements epsilon-greedy algorithm
Parameters:
    Epsilon: Epsilon value
    Steps: Number of steps
    Arms: A vector with the reward values for each arm
    Generator: Type of random number generator being used
Outputs:
    Reward: Vector with reward values for each iteration
    TotalReward: Vector with the sum of rewards
"""
import numpy as np
import math
from matplotlib import pyplot as plt
import random
from random import randint

def EpsilonGreedy(Epsilon, Steps, Arms, Generator):
        global Reward
        global ArmsNumber
        Reward = []
        TotalReward = []
        ActualStep = 1
        ArmsNumber = len(Arms)
        RandomPlace = np.random.rand()
        for N in range(ArmsNumber, 0, -1):
                if RandomPlace >= (N/ArmsNumber):
                        Place = N
                        break
                else:
                        Place = N - 1
        Reward.append(Arms[Place])
        TotalReward.append(sum(Reward))
        while ActualStep < Steps:
                if Generator == 'Random':
                        Reward = RandomGenerator(Epsilon, Arms)
                        TotalReward.append(sum(Reward))
                        ActualStep += 1
        return Reward, TotalReward

def RandomGenerator(Epsilon, Arms):
        global Reward
        global ArmsNumber
        Random = np.random.rand()
        if Random >= (1 - Epsilon):
                Reward.append(max(Reward))
        else:
                RandomPlace = np.random.rand()
                for N in range(ArmsNumber, 0, -1):
                        if RandomPlace >= (N/ArmsNumber):
                                Place = N
                                break
                        else:
                                Place = N - 1
                                Reward.append(Arms[Place])
        return Reward

z , x = EpsilonGreedy(0.1413, 21, [0,0,0,1], 'Random')
y = np.linspace(0, 21, 21);
ax = plt.plot(y, x)
plt.show()
```
